# Non-HDL-cholesterol as valid surrogate to apolipoprotein B_100 _measurement in diabetes: Discriminant Ratio and unbiased equivalence

**DOI:** 10.1186/1475-2840-10-20

**Published:** 2011-02-28

**Authors:** Michel P Hermans, Frank M Sacks, Sylvie A Ahn, Michel F Rousseau

**Affiliations:** 1 Endocrinology and Nutrition Unit, Université catholique de Louvain, 10 Avenue Hippocrate, Brussels (1200), Belgium; 2 Nutrition Department, Harvard School of Public Health, 665 Huntington Avenue, Boston (Ma 0215), USA; 3 Division of Cardiology, Université catholique de Louvain, 10 Avenue Hippocrate, Brussels (1200), Belgium

## Abstract

**Background:**

Apolipoprotein B_100 _(apoB) is a superior indicator of CV risk than total or LDL-C. Non-HDL-C represents a simple surrogate for apoB in hypertriglyceridemic and/or T2DM patients. ApoB and non-HDL-C show high correlation, although the degree of mutual concordance remains debated in CV risk evaluation.

**Objectives:**

We used the *Discriminant Ratio *(DR) methodology to compare the performance of non-HDL-C with that of apoB to rank diabetic patients according to dyslipidemia and to establish the underlying relationship between these variables taking measurement noise and intra-/intersubject variation into account, and to derive an unbiased equivalence equation.

**Methods:**

Fasting total C, HDL-C, apoB and triglycerides were measured in 45 diabetic patients. The DR of the underlying between-subject standard deviation (SD) to the within-subject SD was calculated from duplicates. Correlation coefficients between pairs were adjusted to include an estimate of the underlying correlation.

**Results:**

Mean values [day 1 (1SD)] were 143 (36) mg/dl (non-HDL-C) and 98 (24) mg/dl (apoB). The DR's of both parameters were similar (1.76 and 1.83) (p = 0.83). Pearson's product-moment correlation coefficient between tests was very high (0.94), reaching unity (1.00) after attenuation adjustment. The unbiased equation of equivalence relating apoB to non-HDL-C had a slope of 0.65 and an intercept of 6.3 mg/dl.

**Conclusions:**

The discrimination power of non-HDL-C is similar to that of apoB to rank diabetic patients according to atherogenic cholesterol and lipoprotein burden. Since true correlation between variables reached unity, non-HDL-C may provide not only a metabolic surrogate but also a candidate biometrical equivalent to apoB, as non-HDL-C calculation is readily available.

## Introduction

Total cholesterol and low-density lipoprotein cholesterol (LDL-C), the major component of the former, represent key standard modifiable risk factors for atherosclerotic cardiovascular disease. The hallmark of atherogenic dyslipidemia is low levels of high-density lipoprotein cholesterol (HDL-C) as well as elevated triglycerides (TG) levels, while LDL-C level may only be marginally elevated in this setting [1,2]. A single apolipoprotein B_100 _(apoB) molecule is present in all major atherogenic particles of liver origin (very-low and intermediate-density lipoproteins (VLDL and IDL), and LDL). Therefore, measurement of apoB provides direct information on the number of atherogenic particles (LDL and non-LDL), irrespective of their size. Further, these atherogenic particles, collectively known as non-HDL lipoproteins, are associated with atherogenic dyslipidemia, insulin resistance, portal hyperinsulinemia and the metabolic syndrome phenotype [3-11]. Various ratios were introduced to increase epidemiological prediction of cholesterol, and include the ratio of total or LDL-C to HDL-C, and that of apoB to apoA-I, which represents the ratio of the level of prograde, liver-derived, cholesterol-transporting, major lipoprotein divided by the level of retrograde, reverse cholesterol-transporting lipoprotein [12-15].

Although establishing this latter ratio does not require fasting conditions, these apolipoproteins are not currently part of routine laboratory lipid assessment. Yet, it is now established that both apoB measurement and non-HDL-C (often considered a surrogate of the former, and easily obtained from subtracting HDL-C from C) are better predictors of CVD that LDL-C (even when the latter is directly measured instead of derived from Friedewald's formula), evidence which should eventually lead to some revision of the current risk paradigm [3-11].

Non-HDL-C was introduced as another means to refine risk estimation beyond LDL-C from Friedewald's formula in the presence of raised triglycerides (TG) levels (≥200 mg/dl), since associated changes in VLDL-TG/VLDL-C ratio may lead to LDL-C undercalculation [2]. As it actually estimates the level of all apoB-carrying lipoproteins, non-HDL-C may represent a simple and inexpensive surrogate to apoB measurement, especially in selected patients groups, such as hypertriglyceridemic patients and/or patients with diabetes. The current debate focuses on which of the two should be determined, and in which specific subgroups. It was suggested that apoB might be more performant in identifying patients at CVD risk in the low-risk range and/or in normotriglyceridemic patients, whereas non-HDL-C may be more appropriate in higher-risk patients or in the setting of elevated triglycerides levels or cardiometabolic states [3-5,7,16-18].

ApoB and non-HDL-C measurement show high correlation, although mutual concordance remains a subject of debate in CV risk evaluation. While correlation between apoB and non-HDL-C is reckoned very high, the issue of concordance, agreement and equivalence of use for predicting risk in diabetic patients remains controversial [3-5]. The present study uses the *Discriminant Ratio *(DR) methodology developed by Levy *et al*. to compare the performance of non-HDL-C to that of apoB to rank diabetic patients according to spread of individual lipid values, from normal to dyslipidemia [19-21]. Levy *et al*. methodology standardises comparisons of imprecise tests by taking into account fundamental properties for assessing imprecision and practical performance of tests designed to measure similar physiological variables. This three-steps comparison approach requires (*i*) establishing a discriminant ratio and relative discriminant power; (*ii*) determining the maximum expected rank correlation with another test taking measurement noise into account; and (*iii*) defining an unbiased line of equivalence relating non-HDL-C to apoB.

## Methods and statistical analysis

We studied 45 consecutive North-Caucasian subjects with diabetes mellitus (type 1; *n *= 23 and type2; *n *= 22) and a wide range of atherogenic cholesterol and atherogenic particles number values, from normal to various degrees of (un)treated dyslipidemia, representing a practical range of lipid values as observed in clinical cardiology or diabetes settings.

All lipid values were obtained in the fasting state on 2 different, non-consecutive days. The time-span between samples was 1-3 months, as they were taken during regular outpatients follow-up visits, with lipid-lowering drugs neither prescribed, introduced, switched nor titrated between duplicate samplings. Total cholesterol and triglycerides were determined using SYNCHRON^® ^system (Beckman Coulter Inc., Brea, CA). HDL-C was determined with ULTRA-N-geneous^® ^reagent (Genzyme Corporation, Cambridge, MA). ApoB was determined with immunonephelometry on BNII Analyzer^® ^(Siemens Healthcare Products GmbH, Marburg, Germany). The within-subject coefficients of variation were: 6.9% (apoB), 5.4% (total cholesterol), and 7.1% (HDL-C). LDL-C was calculated according to Friedewald's formula [22].

The DR methodology compares different tests measuring the same underlying physiological variable by determining the ability of a test to discriminate between different subjects, and the comparison of discrimination between different tests as well as the underlying correlation between pairs of tests adjusting for the attenuating effect of within-subject variation [19]. In a comparison study where duplicates measurements are performed in each subject, the measured between-subjects standard deviation (SD_B_) is calculated as the SD of the subjects' mean values calculated from the 2 replicates. The standard mathematical adjustment to yield the *underlying *between-subject SD (SD_U_) is: SD_U _= √ (SD^2^_B _- SD^2^_W_/2). The *within*-subject variance (V_W_) is calculated for *m *repeat tests as (V_W_) = S(x_j _-x_i _)^2^/(m-1)), the within-subject SD (SD_W_) being its square root. The DR represents the ratio SD_U_/SD_W_. Confidence limits for DR's and the testing for equivalence of different DR's were calculated and differences were considered significant for *p *< 0.05. Given sample size and number of replicates, the minimal detectable significant difference in DR for the present study was calculated as 0.80. Coefficients of correlation between pairs of tests used to estimate the severity of dyslipidemia (non-HDL-C and apoB) were also adjusted to include an estimate of the underlying correlation, since standard coefficients tend to underestimate the true correlation between tests due to the presence of within-subject variation [19].

## Results

The patients' characteristics are described in Table 1. Patients' mean age (1 SD) was 52 (16) years, sex ratio (M/F) was 63/37, BMI was 26.7 (3.4) kg/m^2^, and diabetes duration 15 (11) years. Eighty-three percent of patients were in primary macrovascular prevention. Mean glycaemic control, as reflected by current HbA_1c_, was suboptimal at 7.9 (1.6)% (normal value: 4.0-6.0%). Thirty-one percent of patients were treated with statins and/or fibrates.

**Table 1:** Patients' characteristics

*N*		*45*
**age**	*years*	**52.1 **(16.2)
**sex ratio (M : F)**	*%*	**63 : 37**
**BMI**	*kg.m*^*-2*^	**26.7 **(3.4)
**diabetes duration**	*years*	**15.0 **(11.4)
**HbA**_**1c**_	*%*	**7.9 **(1.6)
**systolic and diastolic BP**	*mm Hg*	**133 **(17) - **78 **(9)
**macroangiopathy (CAD &/or PAD)**	*%*	**17**
**statin &/or fibrate therapy**	*%*	**31**
**total cholesterol**	*mg.dL*^*-1*^	**199 **(38)
**LDL-cholesterol**	*mg.dL*^*-1*^	**116 **(31)
**HDL-cholesterol**	*mg.dL*^*-1*^	**56 **(17)
**non-HDL-cholesterol**	*mg.dL*^*-1*^	**143 **(36)
**apolipoprotein B**_**100**_	*mg.dL*^*-1*^	**98 **(24)
**triglycerides**	*mg.dL*^*-1*^	**135 **(90)

Results are expressed as means (1 SD) or as proportions (%). BMI: body mass index; BP: blood pressure; CAD: coronary artery disease; PAD: peripheral artery disease.

Mean lipid values (day 1) are illustrated in Table 1. In the combined group, the respective range [percentile 25-75] for non-HDL-C was 94-284 [117-160] mg/dl, while the range for apoB was 59-183 [85-113] mg/dl. In T1DM (*n *= 23), mean non-HDL-C was 131 (29) mg/dl, and apoB 91 (21) mg/dl, whereas in T2DM (*n *= 22), mean non-HDL-C was 155 (38) mg/dl, and apoB 106 (25) mg/dl. There were significant differences regarding both parameters between T1DM and T2DM (p = 0.0214 (non-HDL-C) and p = 0.0345 (apoB)). On average, T2DM patients had higher non-HDL-C values (absolute increase 24 mg/dl, i.e. +18%), whereas they also had increased apoB level (absolute increase 15 mg/dl, i.e. +16%).

**Figure 1:**
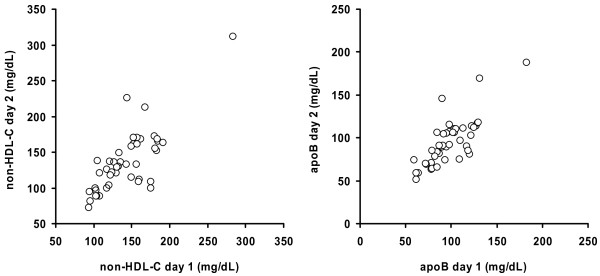
**Plots of untransformed values obtained on day 1 (*X axis*) and day 2 (*Y axis*) for non-HDL-cholesterol (non-HDL-C; *left panel*) and apolipoprotein B_100 _(ApoB; *right panel*) ratios in *n *= 45 subjects with diabetes mellitus**.

Figure 1 illustrates the plots of non-HDL-C and apoB values (day 1 *vs. *day 2), and the clinical range of lipid values obtained from the combined T1DM and T2DM groups. The figure also confirms the homoscedastic behaviour on repeat testing of the data spread.

The SD_U_, SD_W _and DR's of non-HDL-C and apoB are shown on Table 2 for T1DM, T2DM and the combined T1DM and T2DM group. In all three analyses, the DR's of both lipid parameters were almost similar (1.87 vs. 2.08 (T1DM); 1.53 *vs*. 1.51 (T2DM) and 1.76 and 1.83 (all patients), and the observed difference in discriminatory power did not reach statistical significance (*p *= 0.7052 (T1DM); *p *= 0.9710 (T2DM), and *p *= 0.8336 (all patients), respectively).

**Table 2:** Tests precision and discrimination expressed as *underlying between-subject Standard Deviation *(SD_U_), *global within-subject Standard Deviation *(SD_W_), and test *Discriminant Ratio *(DR)

	**SD**_**U**_	**SD**_**W**_	DR	[CIs]
**T1DM **(*n *= 23)				
**non-HDL-C**	**27.8**	**14.9**	**1.87**	[1.30 - 2.78]
**apoB**_**100**_	**21.1**	**10.2**	**2.08**	[1.48 - 3.06]
**T2DM **(*n *= 22)				
**non-HDL-C**	**36.0**	**23.5**	**1.53**	[1.00 - 2.36]
**apoB**_**100**_	**21.1**	**14.0**	**1.51**	[0.99 - 2.34]
**All patients **(*n *= 45)				
**non-HDL-C**	**34.4**	**19.6**	**1.76**	[1.35 - 2.33]
**apoB**_**100**_	**22.3**	**12.2**	**1.83**	[1.41 - 2.42]

Values from log results of individual tests and means of their duplicates in 45 patients. Confidence intervals [CIs] for DR's [2.5-97.5%] in square brackets. apoB_100_: apolipoprotein B_100_; C: cholesterol; HDL: high density lipoprotein; SD: standard deviation; T1DM and T2DM : type 1 and type 2 diabetes mellitus.

**Figure 2:**
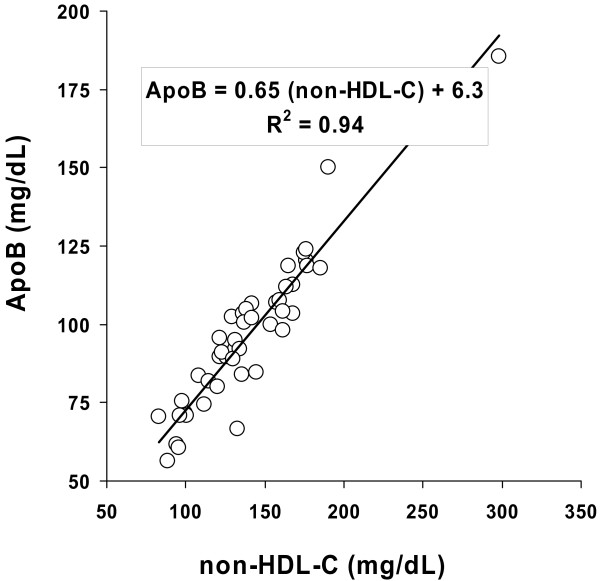
**Plots of means of duplicate measurements obtained on day 1 and day 2 for non-HDL-cholesterol (non-HDL-C; *X axis*) and apolipoprotein B_100 _(ApoB; *Y axis*) in *n *= 45 subjects with diabetes mellitus. **The line represents the *uncorrected linear regression *equation relating non-HDL-C and apolipoprotein B_100 _[ApoB = 0.60 (non-HDL-C) + 12]. The *insert box *provides the *unbiased equivalence equation *which calculates ApoB from non-HDL-C based on the discriminant ratio methodology. *R*^*2 *^represents the measured Pearson correlation coefficient between tests, prior to attenuation adjustment.

The measured Pearson's product-moment correlation coefficient between the two tests was very high (0.94), reaching unity (1.00) once values were correlated after adjustment for attenuation. The plots of the duplicate means for non-HDL-C and apoB on day 1 and day 2 are shown for all patients on Figure 2.

The *uncorrected linear regression equation *relating non-HDL-C and apolipoprotein B_100 _was:

apoB (mg/dl)=0.60 [non−HDL−C(mg/dl)]+12.0 mg/dl

Whereas the *unbiased equation of equivalence *based on the discriminant ratio methodology relating the two measurements was:

apoB (mg/dl)=0.65 [non−HDL−C(mg/dl)]+6.3 mg/dl

## Discussion

This study demonstrates that in diabetic patients, non-HDL-C and apolipoprotein B_100 _performed equally well to discriminate patients according to their atherogenic cholesterol values or atherogenic particles number. In addition, as the underlying correlation between these two continuous variables reached unity once attenuation was taken into account, these two measurements may be used to assess what represents an equivalent underlying biological condition, and that they can substitute for each other for that respect.

While it is well-recognized that non-HDL-C and apoB are closely related metabolically, yet there is ongoing discussion as to whether one should be measured preferentially over the other, with some considering apoB as a choice proatherogenic index in patients with cardiometabolic risk associated with atherogenic dyslipidemia. Our results confirm the equivalence of both measurements in their capacity to rank diabetic patients with a broad spectrum of lipid values, from normal to frank dyslipidemia with elevated atherogenic cholesterol (non-HDL-C) and/or atherogenic particles number (apoB).

In this study involving diabetic patients, apoB did not perform significantly better than non-HDL-C to rank patients according to atherogenic dyslipidemia. Noteworthy, lack of significant difference in DRs was not a bias caused by accretion dilution from pooling T1DM and T2DM patients with divergent DRs. Whereas non-HDL-C is derived from the comput of two robust, well-established measurements methods (total cholesterol and HDL-C), its DR may be negatively affected by amplification error from the incorporation of the intrinsic imprecisions of two measurements. The small difference (0.07) in DR that we observed in this study may be viewed as not clinically meaningful, and there were no significant differences in discrimination between non-HDL-C and apoB in both T1DM and T2DM subgroups, as well as in the combined group.

Such difference in the DR of non-HDL-C and that of apoB would nevertheless translate into a meaningful statistical superiority in the setting of much larger cohort sizes (i.e. n > 3500 when dealing with duplicate measurements or n > 1800 for triplicates). Thus, the higher DR of apoB may represent one contributor to higher performance of determining atherogenic particles number (apoB) against measuring atherogenic cholesterol level (non-HDL-C) to predict coronary heart disease, as observed by Pischon *et al. *[8]. In a routine clinical setting, usually dealing with individual risk estimation or risk stratification between dozens to hundreds of patients, both non-HDL-C and apoB performed equally well to discriminate between diabetic patients, and may be considered as interchangeable surrogates in diabetes.

In the long run, apoB may eventually supersede non-HDL-C, once a sufficient number of large prospective studies confirm its superiority in risk prediction, and after a consensus is reached regarding between-assay standardization. ApoB determination alleviates the requirement for fasting conditions, as mentioned in INTERHEART [13]. Meanwhile, the DR method allows establishing an unbiased equation of equivalence relating non-HDL-C to apoB in diabetic subjects. Since the former is readily available from routine lipid profile in the fasting state whatever the TG level, this equation alleviates the need to perform additional, and at present more costly, apoB determination. Once attenuation was taken into account, both measurements were related by a line of unity, and had similar performance for ranking diabetic subjects according to the risk afforded by apoB-containing particles in scope with the current cholesterol hypothesis paradigm. As both non-HDL-C and apoB represent essentially the same underlying biological variables in diabetics, both can therefore be substituted to each other once an unbiased equation of equivalence is used.

This study has several potential limitations. An easily refutable one is sample size, as the DR methodology does not require large samples, with *n *≥ 20 for 2 replicates being adequate as long as sample represents patients spread over a meaningful clinical range for the variables under study [19]. As the majority of T1DM are normolipaemic, combining T1DM and T2DM patients with significant differences in both non-HDL-C and apoB, as well as patients with and without lipid-lowering drug(s), generated an optimal spread of atherogenic lipids and particles number, as required by the DR methodology for assessing the performance of a continuous physiological variable. In contrast, the reported observations need to be verified in other non-diabetic populations with various severities of (un)treated dyslipidemia. A second limitation is that postprandial lipids were not compared, and the conclusions may not necessarily apply to this state. Yet, neither non-HDL-C nor apolipoprotein B_100 _levels are substantially affected by postprandial excursions in TG-rich lipoproteins, the latter arising mostly from *de novo *secretion of gut-derived apolipoprotein B_48_-containing chylomicrons.

Although non-HDL-C is derived from two separate biological assays (total cholesterol and HDL-C) each with respective imprecision and day-to-day variation, the DR methodology demonstrates that the discrimination of non-HDL-C was similar to that of apoB, measured from a single assay. Non-HDL-C translates into biological measurement of both the number (since it mostly estimates cholesterol from apoB-carrying lipoproteins) and the combined cholesterol mass of atherogenic apoB-containing particles. Non-HDL-C determination includes cholesterol from atherogenic lipoproteins not captured by Friedewald's estimation, such as apoB-carrying remnants not belonging to LDL or IDL and often found in diabetic patients and/or in subjects with metabolic syndrome [14,16,18]. The high agreement that we observed between methods in this population may also reflect bidirectional shifts in the distribution of apoB-containing particles size and cholesterol content seen in diabetic states, including concomitant increases in TG-poor (such as small-dense LDL), as well as TG-rich particles.

In parallel to determination of circulating apolipoproteins, lipid ratios, still widely reported on lab reports, incorporate measurements of total or atherogenic lipids (total C, LDL-C, apoB as *numerator*) and measurements of HDL-C or apoA-I to evaluate reverse cholesterol transport particles as *denominator*. We previously reported that with respect to ratio-based lipid markers, the highest DR's were those of total C/HDL-C and non-HDL-C/HDL-C, which were significantly better than estimated LDL-C/HDL-C. In addition, the apoB/apoA-I ratio had a non-significantly higher DR than non-HDL-C and estimated LDL-C/HDL-C [14]. Due to physiopathological relevance, robustness, ease and low-cost, and imperviousness to fasting conditions, the discriminant ratio of non-HDL-C/HDL-C renders it suitable for routine ranking of atherogenicity in a given patient with type 2 diabetes and/or insulin resistance, exposed to high number of LDL (including small and dense ones), VLDL and remnants lipoproteins.

With respect to LDL-C and non-LDL-C therapeutic targets, a recent joint Consensus Statement (*American Diabetes Association *and *American College of Cardiology Foundation*) suggests new treatment goals for apoB in patients with atherogenic dyslipidemia and cardiometabolic risk. An apoB level <90 mg/dL was proposed in patients without diabetes or known CVD but with ≥2 additional major CVD risk factors, or with diabetes and without major CVD risk factors. An apoB level <80 mg/dL was suggested for patients with the highest CVD risk, i.e. known CVD or diabetes plus ≥1 additional major CVD risk factor [18]. With respect to other determinations, there is growing evidence suggesting that direct measurements of other specific apolipoproteins may contribute to refine CV risk assessment. Thus, apolipoprotein CIII (apoCIII) level may capture a sizeable component of TG-attributable CV risk, and apoCIII measurement may be suited to estimate aspects of hepatic production of a highly-atherogenic VLDL subset and of their derived atherogenic particles, although at the moment apoCIII measurement remains in the realm of research [23-25].

In conclusion, the present study demonstrates, using the validated DR methodology, that the discrimination of non-HDL-C is similar to that of apoB in diabetic patients. Non-HDL-C represents not only a metabolic surrogate, but is close to a true biological equivalent of apoB in this specific population. Besides its original usefulness for estimating LDL-C atherogenicity in hypertriglyceridemic patients with TG values outside of Friedewald's formula's range, non-HDL-C is an easy and cost-effective means to estimate apoB levels, while waiting for a consensus whether to use apoB (*i*) as an alternative to LDL-C for biological assessment of hypercholesterolemia, (*ii*) as a residual risk assessment tool in dyslipidemia, or (*iii*) as secondary therapeutic target beyond routine lipids measurement [26-28].

## Competing interests

The authors declare that they have no competing interests.

## Authors' contributions

All authors read and approved the final manuscript: MPH collected the and managed the T2DM patients' database; MPH, FMS, SAA and MFR contributed equally to the study design, data and statistical analyses, and to drafting the manuscript.
